# Enhanced Sensitivity of a Hydrogen Sulfide Sensor Based on Surface Acoustic Waves at Room Temperature

**DOI:** 10.3390/s18113796

**Published:** 2018-11-06

**Authors:** Xueli Liu, Wen Wang, Yufeng Zhang, Yong Pan, Yong Liang, Junhong Li

**Affiliations:** 1 Institute of Acoustics, Chinese Academy of Sciences, Beijing 100190, China; liuxueli@mail.ioa.ac.cn (X.L.); zhangyufeng@mail.ioa.ac.cn (Y.Z.); liangyong@mail.ioa.ac.cn (Y.L.); ljh@mail.ioa.ac.cn (J.L.); 2 School of Electronic, Electrical and Communication Engineering, University of Chinese Academy of Sciences, Beijing 100190, China; 3 State Key Laboratory of NBC Protection for Civilian, Beijing 102205, China

**Keywords:** surface acoustic wave (SAW), hydrogen sulfide (H_2_S), triethanolamine (TEA), phase discrimination, room temperature

## Abstract

In this contribution, a new surface acoustic wave (SAW)-based sensor was proposed for sensing hydrogen sulfide (H_2_S) at room temperature (30 °C), which was composed of a phase discrimination circuit, a SAW-sensing device patterned with delay line, and a triethanolamine (TEA) coating along the SAW propagation path of the sensing device. The TEA was chosen as the sensitive interface for H_2_S sensing, owing to the high adsorption efficiency by van der Waals’ interactions and hydrogen bonds with H_2_S molecules at room temperature. The adsorption in TEA towards H_2_S modulates the SAW propagation, and the change in the corresponding phase was converted into voltage signal proportional to H_2_S concentration was collected as the sensor signal. A SAW delay line patterned on Y-cut quartz substrate with Al metallization was developed photographically, and lower insertion and excellent temperature stability were achieved thanks to the single-phase unidirectional transducers (SPUDTs) and lower cross-sensitivity of the piezoelectric substrate. The synthesized TEA by the reaction of ethylene oxide and ammonia was dropped into the SAW propagation path of the developed SAW device to build the H_2_S sensor. The developed SAW sensor was characterized by being collecting into the phase discrimination circuit. The gas experimental results appear that fast response (7 s at 4 ppm H_2_S), high sensitivity (0.152 mV/ppm) and lower detection limit (0.15 ppm) were achieved at room temperature. It means the proposed SAW sensor will be promising for H_2_S sensing.

## 1. Introduction

As a toxic combustible gas with peculiar odor, hydrogen sulfide (H_2_S) may cause acute poisoning and chronic, which will damage the central nervous and respiratory system and stimulate the mucous membrane, and even cause death [1]. Therefore, rapid, accurate, and sensitive detection and monitoring towards H_2_S is an essential way to protect against the above issues.

**Figure 1:**
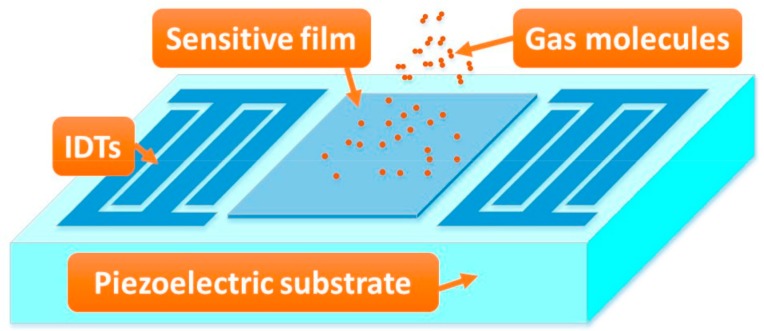
The schematic of the surface acoustic wave (SAW) gas sensor.

The available approaches for sensing H_2_S include metal oxide sensors, spectroscopy sensors, and electrochemical sensors. Among them, the metal oxide sensor realizes the detection of H_2_S physically or chemically by the gas adsorption on the surface of the metal semiconductor, changing the resistance of the semiconductor. Such a sensor appears to have a fast response and high sensitivity, but still suffers from high working temperature to play the catalytic role, which induces more power and a weak working life. Based on the spectral characteristics of the species, the spectroscopy sensor features high precision and satisfactory stability, but it is bulky and costly. Electrochemical sensors detect H_2_S by measuring the potential or current proportional to the gas concentration. There are still some problems in terms of response speed, sensitivity, and selectivity. Compared with available H_2_S gas sensing technology, surface acoustic wave (SAW)-based gas sensors have attracted more attention because of its high sensitivity, fast response, and small volume. A typical SAW gas sensor described in Figure 1 consists of a SAW device patterned by a delay line, and a selective gas-sensitive thin-film coated along the SAW propagation path between the two interdigital transducers (IDTs). The adsorption in sensitive thin-film modulates the SAW propagation by a so-called mass loading or viscoelastic or acoustic–electric effect, depending on the physical class of the sensitive thin-film itself. Considering the photo-lithographical fabrication technique and sensing topological structure, the SAW gas sensor exhibits some unique advantages, such as its low cost, fast response and high sensitivity, which was reported successfully for sensing H_2_, CH_4_, NO_2_, SO_2_ and various chemical agents since the pioneering work of Whltjen et al. [2,3,4,5,6,7,8,9,10]. Among them, some SAW gas sensors can detect gases such as NO_2_ [11], CH_4_ [12], and C_2_H_6_O [13] at room temperature. Obviously, SAW technology provides an effective way for H_2_S detection by choosing an available sensitive material. The present sensitive interfaces for sensing H_2_S are mainly tin oxide (SnO_2_) and their derivatives, which appears to have a high sensitivity and fast response. However, similar to the metal oxide sensor described above, it still suffers from a high working temperature up to 400 °C [14,15,16,17,18]. Hence, exploration in new gas-sensitive materials is the main topic in the investigation of SAW H_2_S sensors.

A triethanolamine (TEA) was chosen as the gas-sensitive material in this work to improve the gas-sensing properties of the SAW H_2_S sensor [19]. The TEA was synthesized by the reaction of ethylene oxide and ammonia and dissolved in the methanol, and dropped onto the SAW propagation path of the photographically fabricated SAW-sensing device with delay line pattern. The viscoelastic nature of the TEA itself was addressed by measuring the changes in frequency response of the sensing device with various TEA thicknesses. The prepared TEA-coated sensing device was connected into the phase discrimination circuit to build the SAW H_2_S sensor, and corresponding converted voltage signal was collected to characterize the H_2_S to be detected. The proposed SAW sensor was exposed to various concentration of H_2_S at room temperature (30 °C), and the resulting performance, measured in terms of sensitivity, temperature stability, detection limit, and repeatability was characterized experimentally.

## 2. Technique Realization

### 2.1. SAW Devices

**Figure 2:**
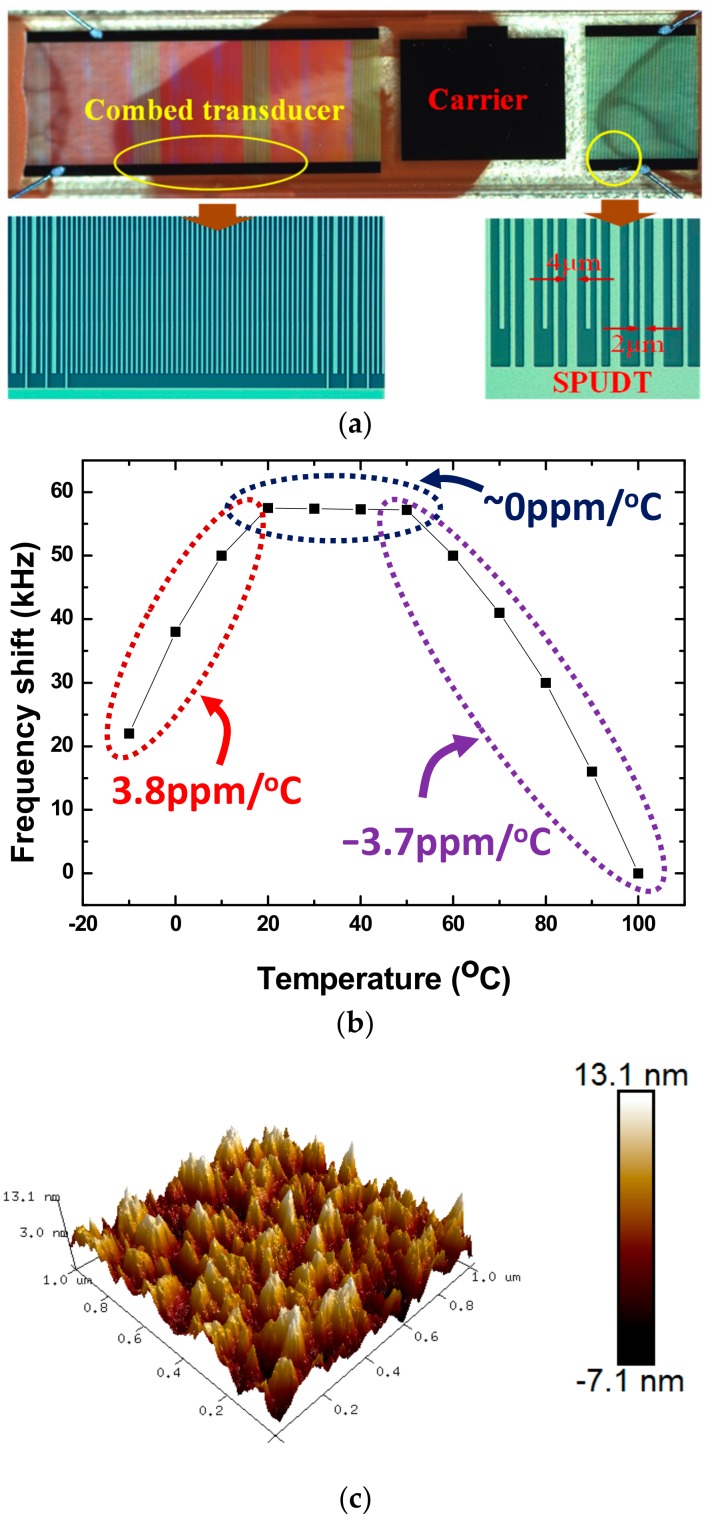
(**a**) The optical picture of the prepared SAW device for H_2_S sensing, (**b**) measured crossed temperature sensitivity of the proposed SAW device, and (**c**) the Atomic Force Microscop (AFM) picture of the TEA coating.

The SAW delay line pattern was used for the proposed SAW H_2_S sensor, which was consists of two photo-lithographically defined Aluminum (Al) interdigital transducers (IDTs) separated by a path length of 2 mm fabricated on Y-cut quartz substrate with excellent temperature stability. Single-phase unidirectional transducers (SPUDTs) described in Figure 2, confining the acoustic energy predominantly in one direction on the piezoelectric substrate surface, were used to form the transducers to reduce the insertion loss [20]. Also, a combed transducer was used to structure the left transducer in Figure 2 to improve phase properties in the pass-band of the device. The operation frequency is designed to operate at 200 MHz, and corresponding wavelength λ is ~15.8 μm. The electrode widths in SPUDTs are ~4 μm (λ/4) and ~2 μm (λ/8), respectively. The lengths of the two transducers of the SAW devices are set to 234 λ and 72 λ, respectively. Aluminum with thickness of 150 nm was deposited on the cleaned Y-cut quartz substrate surface using a thermal evaporator. Then, a 1-mm-thick photoresist (PR) was spin-coated, exposed, and developed for the delay line patterns. Al was wert-etched and PR was dissolved in acetone. Several rinses with DI water were performed to remove any unwanted products. After preparation of the Al electrodes, a 50 nm SiO_2_ thin-film was overlaid to the transducers to provide a good protect in process of gas-sensitive film coating. Finally, the piezoelectric wafer with SAW device pattern was dicing-sawed for wafer bonding and packaging.

**Figure 3:**
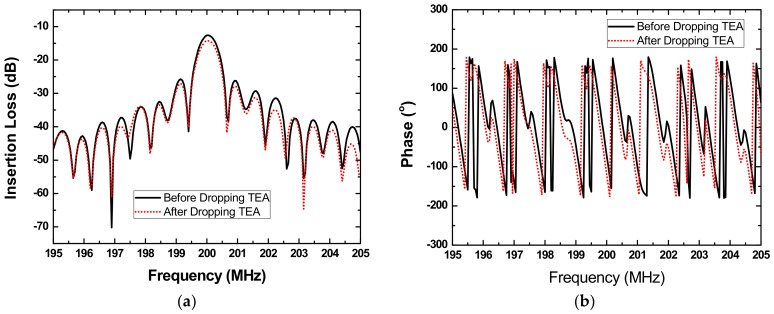
The measured insertion loss (**a**) and phase (**b**) of the proposed sensing device before and after 0.2 μL TEA deposition.

Figure 2a shows the optical picture of the prepared SAW-sensing device with an operation frequency of 200 MHz. The Al thin-film between the two IDTs was used as the carrier for gas-sensitive film deposition. The prepared SAW device was characterized by using the network analyzer as shown in Figure 3a,b, low insertion loss of ~12 dB and linear phase property in the pass-band were achieved.

Also, the crossed temperature sensitivity of the proposed SAW device was tested by using the incubator, as shown in Figure 2b. A very low frequency shift upon temperature was achieved owing to the weak coefficient of thermal expansion of the Y-cut quartz itself, especially almost zero crossed temperature sensitivity was observed in 20–60 °C, which was benefit for improvement of the sensor stability.

### 2.2. TEA Preparation

**Figure 4:**
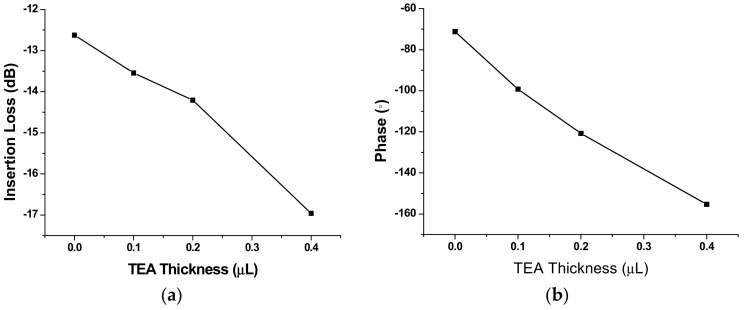
The induced changes in insertion loss (**a**) and phase (**b**) by various TEA thickness.

The TEA was synthesized by the reaction of ethylene oxide and ammonia in a ratio of 1:2. Then the synthesized TEA was dissolved into the methanol, and dropped onto the carrier of the SAW-sensing device. Detail procedure is depicted below. First, a TEA solution with solvent of methanol and concentration of 2.45 mg/mL was prepared. A 1-μL micro-injector was used to drop the TEA solution to the carrier of the sensing device. Various TEA thicknesses can be realized by controlling the dropping times. Thereafter, it was left at room temperature for 24 h to sufficiently release the solvent in the solution. Figure 2b gives the Atomic Force Microscop (AFM) image of a prepared TEA film depositing on the sensing device. Uneven surface was observed because of the inhomogeneity of the drop-coating, which helps enlarge the contract area in gas adsorption and leads to sensitivity improvement. Usually, the TEA deposition will induce obvious acoustic attenuation because of its viscoelastic nature [21]. The prepared TEA-coated sensing device was characterized by using the network analyzer as shown in Figure 3a,b. Obviously, increasing insertion loss of the sensing device was observed with increases in TEA thickness, as well as the changes in phase and frequency response of the sensing device. The induced changes in insertion loss, corresponding phase, and operation frequency are concluded in Figure 4. With increases of dropped amount used, obvious decreases in insertion loss and corresponding phase were observed. The induced insertion loss over 4 dB occurs at 0.4 μL TEA solution to be dropped, which maybe influence the stability of the sensor system. Hence, the TEA solution less than 0.4 μL was used to conduct the SAW sensor in this work.

### 2.3. SAW Sensor System

**Figure 5:**
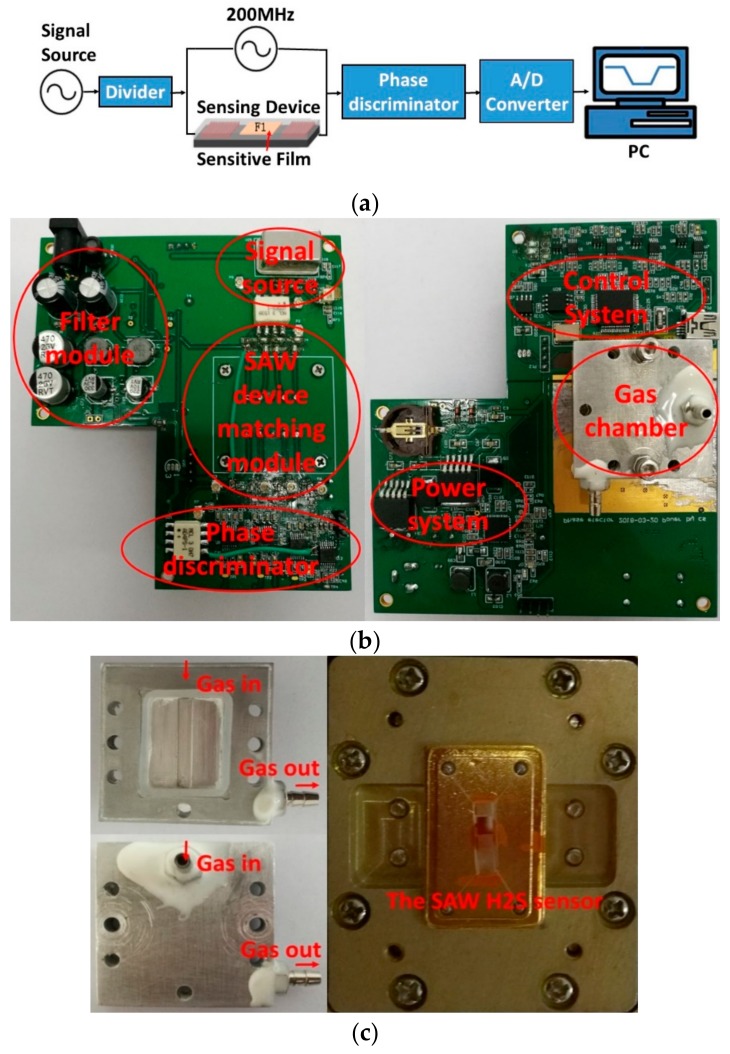

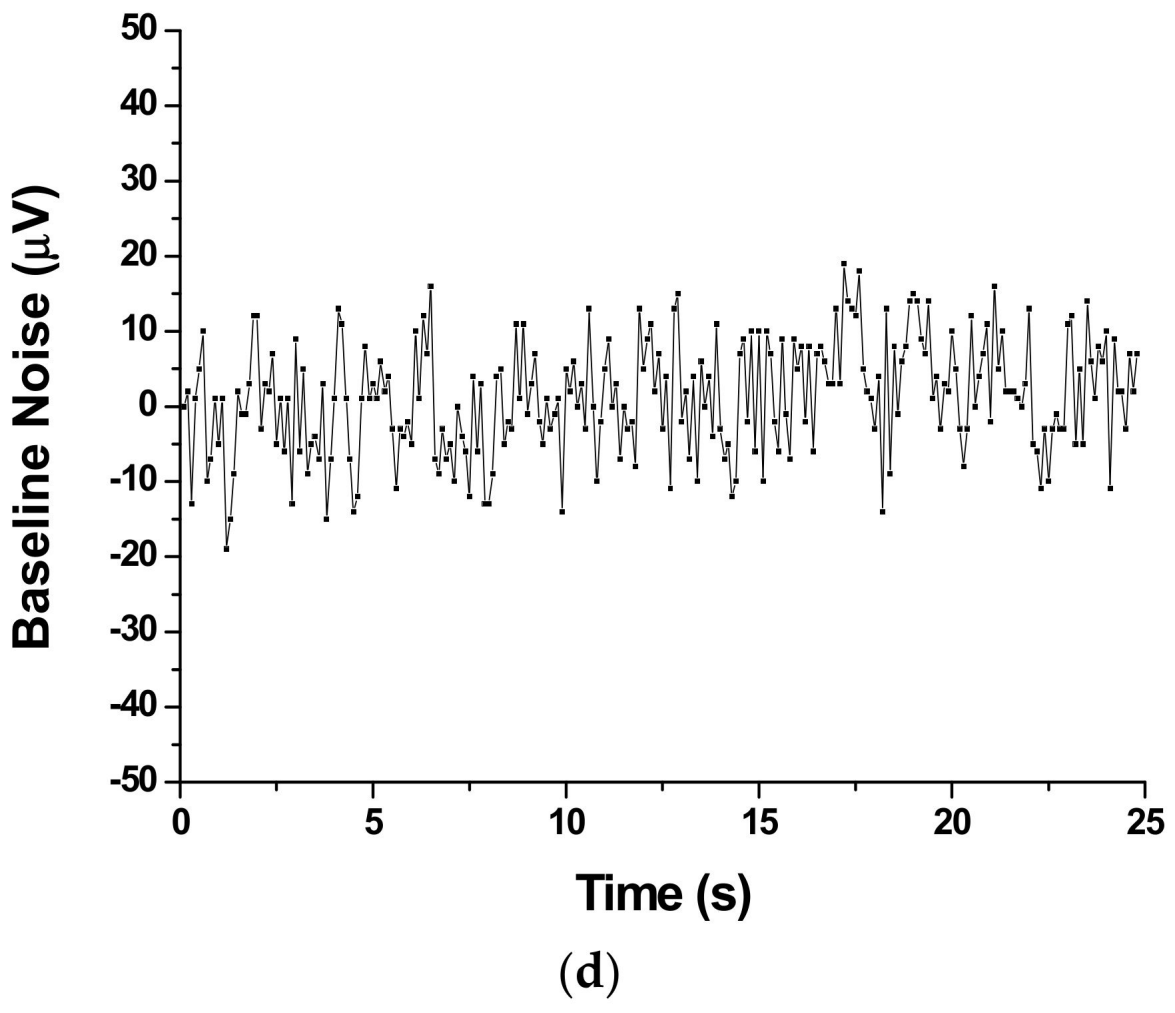
(**a**) The proposed SAW H_2_S sensor system. (**b**) The Printed Circuit Board (PCB) of the sensor system, (**c**) the gas chamber, and (**d**) measured baseline noise.

The prepared TEA-coated SAW-sensing device and naked device acted as the reference were collected into the phase discrimination circuit to build the sensor system, which was composed of the proposed SAW H_2_S sensor, a specific signal source (200 MHz), divider, phase discriminator, A/D converter, and personal computer (PC). The corresponding sensor system was described in Figure 5a. Figure 5b presents the PCB of the sensor system. The prepared sensing devices were embedded into the nickel-plated Al-gas chamber with volume of 500 mL (Figure 5c). Obviously, the baseline noise of the sensor system will affect directly the detection limit and stability of the sensor, which was tested as shown in Figure 5d, excellent stability of ~±20 μV was obtained.

## 3. Experimental Results and Discussions

### 3.1. Experimental Setup

**Figure 6:**
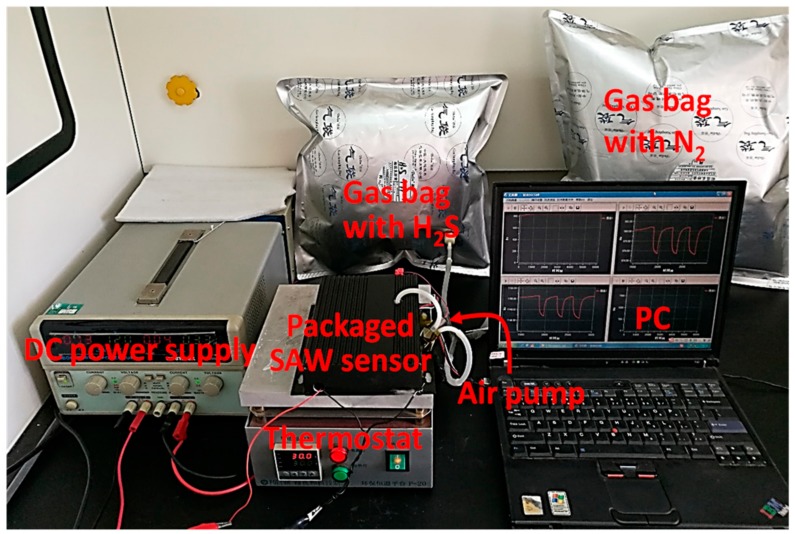
The experimental setup for evaluation of the proposed SAW sensor.

An experimental setup described in Figure 6 was used to evaluate the proposed SAW H_2_S sensor, which includes gas bags (10 L), plastic tube, Y-type shunt tube, air pump (350 mL/min), SAW H_2_S sensor system (Figure 5b), thermostat, PC and DC power supply. In this paper, N_2_ is used as carrier gas to avoid interference from humidity. The gas bag with H_2_S mixed with N_2_ at various concentrations is connected to one branch of the Y-shaped shunt tube through a plastic tube, and the other branches of the Y-shaped shunt are connected to the gas bag with N_2_ acted as the carrier gas and the air pump, and then connected to the SAW sensor. Put the SAW H_2_S sensor system in a metal box in Figure 6 to shield electromagnetic interference. Then place the metal box on the thermostat at 30 °C to maintain a constant ambient temperature. The acquisition and real-time plotting of the sensor signals is conducted by the homebrew software on the PC.

### 3.2. Gas Experiments

**Figure 7:**
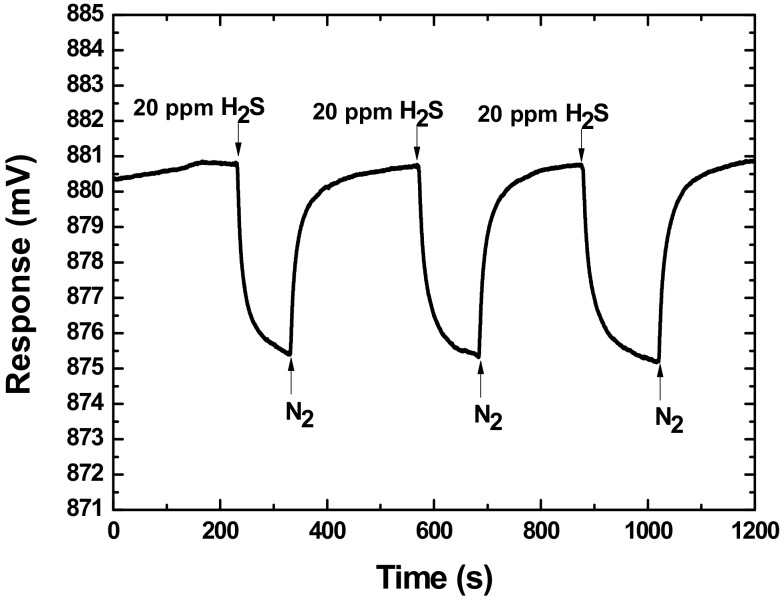
Repeatability test of the proposed SAW sensor at 30 °C.

First, the repeatability of the proposed SAW H_2_S sensor coated with 0.2 μL TEA was evaluated at 30 °C by conduction of three consecutive about 1-min on–off exposures to 20 ppm H_2_S, as shown in Figure 7. We assumed here that response and recovery times corresponded to the times that sensor outputs reached 70% of their final value and 30% off the baseline value, respectively. Three H_2_S exposures are observed in good reproducible run with fast response and larger sensor response listed in Table 1. The gathered sensor signal showed a rapid rise upon exposure to H_2_S and reaches approximately the 70% of equilibrium (saturation) value in 18 s. When the gas was removed by carrier gas injection, the sensor response recovered in less than 22 s to 70% of its initial baseline. It means the 70% response time of ~18 s and 70% recovery time of ~22 s with good repeatability were obtained at 30 °C. These promising results addressed that the proposed SAW sensor exhibits a fast response and excellent repeatability in response to H_2_S.

**Figure 8:**
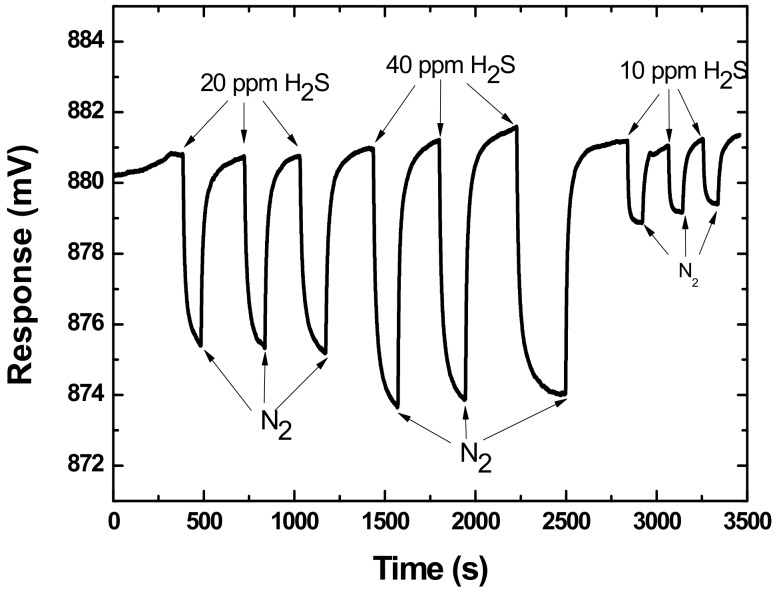
Responses of SAW sensor to different concentrations of H_2_S.

**Figure 9:**
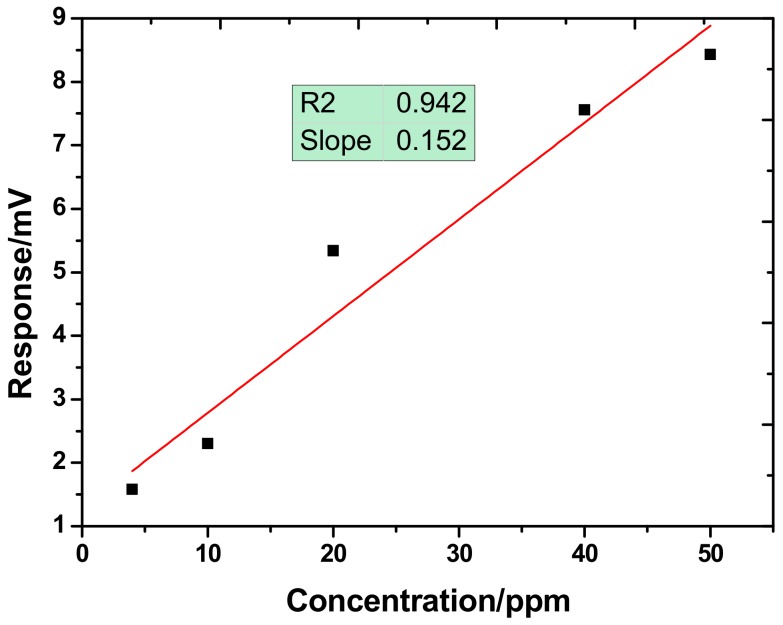
The sensitivity evaluation of the proposed SAW H_2_S sensor.

Next, the proposed SAW sensor coated with 0.2-μL TEA was exposed to H_2_S with various concentrations to characterize their sensitivity. The injected H_2_S gas increases from 4 ppm to 50 ppm. As shown in Figure 8, the sensor has good repeatability in response when exposed to different concentrations of H_2_S. The maximum sensor response was plotted vs. the corresponding H_2_S concentrations as shown in Figure 9. It is obvious that as the gas concentration increased, the sensor signal also increased with approximate linearity. The sensitivity in H_2_S concentration range of 0–50 ppm was evaluated as 0.152 mV/ppm with linearity of 0.942. Also, the picture indicates the response time was related to the concentration of the H_2_S to be detected. For low concentration H_2_S (4 ppm), fast response less than 7s was observed. Also, from the picture, obvious sensor response was achieved with even a very low H_2_S concentration (4 ppm), which means a lower detection limit will be expected because the proposed sensor system produces low baseline noise of ±20 μV. Hence, based on the International Union of Pure and Applied Chemistry (IUPAC) (Zurich, Switzerland), detection limits are calculated as the lowest concentration of analytes giving a signal of three times the baseline noise of the sensor system. Here, a large response of a 1.58 mV voltage shift is found for low concentration of 4 ppm H_2_S from our sensor, indicating very low detection limit of ~0.15 ppm is expected when linear response was assumed at low concentrations.

Upon the experimental results, it can be concluded that the proposed TEA-coated SAW H_2_S sensor features a fast response and high sensitivity at room temperature. Table 2 gives a performance comparison of the proposed SAW sensor in this work with the existing SAW sensor prototypes. Obviously, a faster response and lower detection limit were achieved at room temperature from the proposed SAW sensor.

## 4. Conclusions

In this work, a new SAW sensor was proposed for sensing H_2_S, which was composed of a phase discrimination circuit, a SAW-sensing device patterned with delay line, and a TEA coating along the SAW propagation path of the sensing device. A gas experiment was conducted by using the proposed SAW sensor coated with 0.2 μL TEA exposed upon H_2_S with various concentration from 4 ppm to 50 ppm. And the experimental results appear that fast response (7 s at 4 ppm H_2_S), high sensitivity (0.152 mV/ppm), and a lower detection limit (0.15 ppm) were achieved at 30 °C thanks to the excellent adsorption efficiency of the TEA itself. Obviously, the proposed SAW H_2_S sensor is very promising for actual detection and monitoring of H_2_S.

## Figures and Tables

**Table 1:** Sensor response towards for consecutive exposures to 20 ppm H_2_S.

Response (mV)	Response Time (s)	Recovery Time (s)
5.391	17.3	26.7
5.344	20.5	21.6
5.458	23.8	24.1

**Table 2:** The comparison of the proposed SAW H_2_S Sensor with existing sensor prototypes.

Preparation Method	Lower Limit of Detection (ppm)	Optimum Working Temperature (°C)	Response Time (s)	Reference
Sputtering, pure SnO_2_	1.0375	60	142	[14]
SnO_2_/CuO	0.53	190	30	[15]
Cu NP-SWCNT	5	160	55	[16]
TEA	0.152	30	7	In this work
